# Development of combinatorial antibody therapies for diffuse large B cell lymphoma

**DOI:** 10.3389/fmed.2022.1034594

**Published:** 2022-10-24

**Authors:** Eric S. Geanes, Stacey A. Krepel, Rebecca McLennan, Stephen Pierce, Santosh Khanal, Todd Bradley

**Affiliations:** ^1^Genomic Medicine Center, Children’s Mercy Research Institute, Kansas City, MO, United States; ^2^Department of Pathology and Laboratory Medicine, University of Kansas Medical Center, Kansas City, KS, United States; ^3^Department of Pediatrics, University of Kansas Medical Center, Kansas City, KS, United States; ^4^Department of Pediatrics, University of Missouri-Kansas City, Kansas City, MO, United States

**Keywords:** bispecific antibody, lymphoma, diffuse large B cell lymphoma, rituximab, cancer therapy, CD20, CD74, IL4R

## Abstract

Diffuse large B-cell lymphoma (DLBCL), the most common form of lymphoma, is typically treated with chemotherapy combined with the immunotherapy rituximab, an antibody targeting the B cell receptor, CD20. Despite the success of this treatment regimen, approximately a third of DLBCL patients experience either relapse or have refractory disease that is resistant to rituximab, indicating the need for alternative therapeutic strategies. Here, we identified that CD74 and IL4R are expressed on the cell surface of both CD20 positive and CD20 negative B cell populations. Moreover, genes encoding *CD74* and *IL4R* are expressed in lymphoma biopsies isolated from all stages of disease. We engineered bispecific antibodies targeting CD74 or IL4R in combination with rituximab anti-CD20 (anti-CD74/anti-CD20 and anti-IL4R/anti-CD20). Bispecific antibody function was evaluated by measuring direct induction of apoptosis, antibody-dependent cellular phagocytosis (ADCP), and antibody-dependent cellular cytotoxicity in both rituximab-sensitive and rituximab-resistant DLBCL cell lines. Both anti-CD74/anti-CD20 and anti-IL4R/anti-CD20 were able to mediate ADCC and ADCP, but CD74-targeting therapeutic antibodies could also mediate direct cytotoxicity. Overall, this study strongly indicates that development of bispecific antibodies that target multiple B cell receptors expressed by lymphoma could provide improved defense against relapse and rituximab resistance.

## Introduction

About 4% of all cancer diagnoses in the United States each year are classified as Non-Hodgkin’s lymphomas (NHL) (1). The most common type of NHL is diffuse large B cell lymphoma (DLBCL), which represents 40% of all newly diagnosed lymphomas annually (2). There are many subtypes of DLBCL, which are based on gene expression profiling and location of initiation (3–5). Despite being genetically and phenotypically diverse, most patients with DLBCL are treated with common therapy regardless of the subtypes involved. However, with the advances in immunotherapy, more targeted therapeutic approaches are being implemented.

One such therapeutic is the monoclonal antibody rituximab, which targets the B cell marker CD20 (6). Rituximab was the first monoclonal antibody approved by the United States Food and Drug Administration (FDA) for use in cancer treatment in the late 1990’s, and in the early 2000’s, it was added to the standard DLBCL chemotherapeutic regimen, CHOP, which includes cyclophosphamide, vincristine, doxorubicin, and prednisone (7–13). With the addition of rituximab, R-CHOP demonstrated a 10-year disease-free survival of approximately 64%, an improvement compared to the 42.5% disease-free survival with CHOP treatment alone (8). Despite this success, 30–40% of DLCBL patients experience either relapse or refractory disease with R-CHOP treatment (14–18). There have been various mechanisms proposed for the development of rituximab resistance, including a decrease in complement-dependent cytotoxicity, resistance to killing *via* antibody-dependent cell-mediated cytotoxicity, and resistance to apoptosis (19). One of the most strongly supported hypotheses attributes resistance to the loss of CD20 expression on B cells following initial rituximab treatment (19–21). Importantly, rituximab targets only select subpopulations of B cells and is not a pan-B cell therapeutic (22). For example, plasma B cells do not express CD20 and are not targeted by rituximab. This has led to the development of immunotherapies targeting other B cells markers to have broader clinical application. Due to the phenotypically diverse nature of DLCBL subtypes, it has been suggested that targeting multiple B cell specific pathways or receptors would be a more effective treatment (3). Additionally, development of resistance may prove to be more difficult if there are multiple therapeutic targets, as these targets would need to be simultaneously mutated or expression level reduced to effectively escape treatment (3).

Bispecific antibodies are a new immunotherapy that allows for expression of an antibody that targets two cell surface receptors, simultaneously. There are multiple mechanisms associated with bispecific antibody efficacy, including complement activation; recruitment of macrophages for antibody-dependent cellular phagocytosis (ADCP); recruitment of natural killer (NK) cells or T-cells for antibody-dependent cell-mediated cytotoxicity (ADCC); apoptosis activation; and priming for cross-presentation by antigen-presenting cells (19, 23, 24). One advantage of bispecific antibodies over more conventional treatments is the decreased risk of resistance against two different targets.

In this study, using a bispecific antibody approach, we sought to broaden the therapeutic benefit of rituximab by developing novel lymphoma-targeted bispecific antibodies. We identified CD74 and IL4R as surface receptors that are expressed on B cell populations that have CD20 or do not have CD20 expression, respectively. Moreover, we found that these markers are expressed in lymphomas and could serve as potential therapeutic targets on B cells. We engineered bispecific antibodies against these targets in combination with anti-CD20. We then evaluated these antibodies for functional targeting of lymphoma cell lines.

## Materials and methods

### Single cell RNA sequencing analysis

Single cell RNA sequencing (scRNA-seq) data was acquired from a previously published dataset of scRNA-seq performed on peripheral blood mononuclear cells (PBMCs) from 12 healthy and HIV-infected subjects that were deidentified from Duke University (25). Original sample collection was reviewed and approved by the Duke Medicine Institutional Review Board. The data is publicly available at SRA BioProject ID: PRJNA681021. PBMCs were thawed, washed and placed in single-cell suspensions with PBS + 0.04% bovine serum albumin (BSA). Cellular suspensions were loaded on a GemCode Single-Cell instrument (10X Genomics, Pleasanton, CA, USA) to generate single-cell beads in emulsion. Single-cell RNA-seq libraries were then prepared using a GemCode Single Cell 3′ Gel bead and library kit version 2 (10X Genomics). Single-cell barcoded cDNA libraries were quantified by quantitative PCR (Kappa Biosystems, Potters Bar, UK) and sequenced on an Illumina (San Diego, CA, USA) NextSeq 500 (26–29). Read lengths were 26 bp for read 1, 8 bp i7 index, and 98 bp read 2. Cells were sequenced to greater than 50,000 reads per cell.

After sequencing, the Cell Ranger Single Cell Software Suite (version 2.1.1) was used to generate sequencing FASTQ files and to perform sample de-multiplexing, barcode processing, reference alignment and single-cell 3′ gene counting (30). Reads were aligned to the human genome (GRCH38). Samples were aggregated using the CellRanger Aggr function to create a single matrix of cell barcodes and gene counts for the groups. During the process each library was normalized for mapped sequencing depth. In order to control for variation in the number of reads per sample (sequencing depth), reads were subsampled from higher-depth libraries until they all had an equal number of reads per cell that were confidently mapped to the transcriptome. Finally, in order to control for technical variation and correct for any batch effects we used the Seurat analysis pipeline Multi CCA method to regress out cell-cell variation in gene expression. The union of variable genes across all individual samples were utilized to renormalize the data.

Matrices of cell barcodes and gene counts generated by Cell Ranger were loaded into Seurat R package (v3.2.3) for graph-based cell clustering, dimensionality reduction and data visualization (31–33). We filtered low quality cells that had lower than 200 expressed transcripts and percentage of mitochondrial genes expressed greater than 20% and for the primary cell model we reduced this threshold to greater than 10% mitochondrial genes. We included up to 45 PCA dimensions for the PBMCs and 48 PCA for the primary cell model for downstream graph-based clustering and UMAP visualization. All other parameters we followed the default Seurat recommendations. We then selected cells that were B cells using *CD79A* transcript expression and utilized this subset of cells for further analysis. Genes that correlated with *MS4A1* or *CXCR4* transcript expression were calculated by Pearson correlation and corrected for multiple comparisons using Bonferroni. Graphs and plots were generated using the Seurat and ggplot2 (v3.3.3) R packages and Graphpad Prism version 8 (Graphpad Software, San Diego, CA, USA). The single-cell RNA seq unprocessed reads have been deposited in the NCBI SRA database under the BioProject ID: PRJNA681021. Code and other processed file formats are available from corresponding author/s upon reasonable request.

### Lymphoma panel gene expression assay

Lymphoma cDNA Array I and Array II were purchased from OriGene Technologies, Inc. (Rockville, MD, USA). The 2 panels consisted of cDNA from the following tumor samples: 12 normal, 15 stage IE, 41 stage I, 1 stage IIB, 12 stage IIE, 6 stage II, 1 stage III, 7 stage IV, and 1 N/R. Plates with lyophilized cDNA were warmed to room temperature, centrifuged for 30 s at 1,000 revolutions per minute, and pellets from each well suspended in 30 μl of qPCR master mix. For a single 30 μl reaction, the qPCR master mix consisted of 15 μl of 2x Taqman Fast Advanced Master Mix (Applied Biosystems, Waltham, MA, USA), 1.5 μl of 20x Taqman probe (Thermo Fisher Scientific, Waltham, MA, USA), and 13.5 μl of PCR-grade water (Invitrogen, Waltham, MA, USA). Probes utilized in the assay targeted *IGSF9* (Hs00325279_m1), *CD209* (Hs01588349_m1), *MS4A1* (Hs00544819_m1), *IL4R* (Hs00965056_m1), and *CD74* (Hs00269961_m1). An *18S* probe (Hs03003631_g1) was used as a housekeeping control. Only 1 probe was used per master mix. Following resuspension, plates were vortexed, centrifuged and placed on ice. Plates were loaded into the QuantStudio 12 Flex (Applied Biosystems). After loading, the reactions underwent an initial 50^°^C for 2 min activation followed by a 95^°^C for 10 min pre-soak for 1 cycle. The remaining 42 cycles were performed in two steps: denaturing at 95^°^C for 15 s followed by annealing at 60^°^C for 1 min. Data was analyzed by subtracting the Ct values of *18S* from the Ct value of the target gene. This difference (x) was transformed *via* the function 2^(-x) and plotted on a logarithmic scale to illustrate target gene expression relative to *18S* for each sample.

### Cell culture

SU-DHL-4 (CRL-2957), NU-DUL-1 (CRL-2969), and SU-DHL-8 (CRL-2961) cell lines were purchased from the Non-Hodgkin’s Lymphoma Cell Line Panel at ATCC (Manassas, VA, USA) and maintained in RPMI-1640 medium (Gibco, Grand Island, NY, USA) supplemented with 10% fetal bovine serum (Thermo Fisher Scientific). All cell lines were cultured at 37^°^C under 5% CO_2_. Media was refreshed every 48 h.

### RT-PCR

RNA extraction was performed according to manufacturer protocol using RNeasy Plus Mini Kit with the additional use of Qiashredder from Qiagen (Hilden, Germany). cDNA synthesis was performed using SuperScript™ VILO™ cDNA Synthesis kit and High-Capacity cDNA Reverse Transcription Kit (Thermo Fisher Scientific). RT-PCR was performed using TaqMan Fast Advanced Master Mix (Thermo Fisher Scientific) and the following Advanced Biosystems Taqman Probes: *18S* TaqMan Probe (FAM-MGB) Assay ID: Hs99999901_s1, *MS4A1* TaqMan Probe (FAM-MGB) Assay ID: Hs00544819_m1, *CD74* TaqMan Probe (FAM-MGB) Assay ID: Hs00269961_m1, *IL4R* TaqMan Probe (FAM-MGB) Assay ID: Hs00965056_m1.

### Antibodies

Rituximab was purchased from Creative Biolabs (Shirley, NY, USA) and Invivogen (San Diego, CA, USA). Milatuzumab (TAB-763), Dupilumab (TAB-021ML) were purchased from Creative Biolabs. Bispecific antibodies were produced in collaboration with Creative Biolabs. Expression vectors encoding the antibody heavy and light chain gene sequences were transiently transfected and expressed in HEK293F cells. Secreted antibody was purified by Protein A affinity chromatography, ultrafiltration and then subjected to 0.2-micron sterile filtration. Purified antibodies were stored at PBS, pH 7.4. For sequences used for each heavy and light chain in each of the anti-CD20/anti-CD74 bispecific antibody and anti-CD20/anti-IL4R bispecific antibodies (see Supplementary Data 1). Quality of bispecific antibodies was measured by Creative Biolabs by reducing SDS-PAGE and size exclusion chromatography-HPLC (Supplementary Figure 1). AffiniPure Goat Anti-Human IgG, Fcγ fragment specific (109-005-098) cross-linking antibody was purchased from Jackson ImmunoResearch Laboratories, Inc. (West Grove, PA, USA).

### Enzyme-linked immunosorbent assays

Enzyme-linked immunosorbent assays (ELISAs) were performed using the following antigens and antibodies: recombinant CD20 full length protein (Acro Biosystems, Newark, DE, USA), recombinant CD74 protein (R&D Systems, Minneapolis, MN, USA), recombinant IL4R protein (R&D Systems), human Anti-CD20 antibody (Invivogen), Milatuzumab/Anti-CD74 Antibody (Creative Biolabs), Dupilumab/IL4R monoclonal antibody (Creative Biolabs), bispecific anti-CD20/anti-CD74 antibody (Creative Biolabs), bispecific anti-CD20/anti-IL4R antibody (Creative Biolabs). Antigens were all diluted to 2 μg/mL in 0.1 M sodium bicarbonate and incubated on high-binding plates (Corning Inc., Corning, NY, USA) overnight at 4 degrees. Antibodies were diluted to 33.3 μg/mL in superblock buffer with sodium azide followed by subsequent 1:3 dilutions until a final dilution of 0.565 ng/mL. Secondary Goat anti-human IgG antibody (Jackson ImmunoResearch Laboratories, Inc.) dilutions were done in superblock buffer without sodium azide within range of manufacturer’s recommendations at 1:50,000 dilution. SureBlue Reserve Microwell Substrate (VWR, Radnor, PA, USA) was added and incubated in the dark for 15 min. Immediately after, 0.33 N HCl Acid Stop solution was added to the plate and absorbance was measured at 450 nm.

### Crosslinking apoptosis assay

Cells were seeded at 50,000 cells per well in 24 well dishes. 10 μg/mL of respective antibody and AffiniPure Goat Anti-Human IgG, Fcγ fragment specific cross-linking antibody (Jackson ImmunoResearch Laboratories, Inc.) was added to wells and incubated for 8 h. Cells were collected and incubated in the Muse Annexin V reagent according to the manufacture’s protocol. Total cell death was measured and recorded on the Muse Cell Analyzer (Luminex Corporation, Austin, TX, USA) in reference to crosslinking antibody control wells with the addition of only crosslinking antibody.

### Antibody-dependent cellular cytotoxicity assay

ADCC Bioassay Core Kit (Promega, Madison, WI, USA) was performed using the manufacturer protocol with the addition of SU-DHL-4 (CRL-2957), NU-DUL-1 (CRL-2969), and SU-DHL-8 (CRL-2961) cell lines as target cells purchased from ATCC. ADCC Bioassay Complete Kit (Promega) was performed using the manufacturer protocol with the provided Raji target cells. Antibodies were treated at 1 μg/mL and subsequently diluted at a 1:3 ratio until a final concentration of 0.152 ng/mL. Respective target cells were incubated with antibody and modified Jurkat NFAT-luc FcγRIIIa effector cells for 6 h. Bio-Glo luciferase assay reagent was added to each well and luminescence was measured.

### Antibody-dependent cellular phagocytosis assay

ADCP Bioassay Complete Kit (Promega) was performed using the manufacturer protocol with the provided Raji target cells and modified Jurkat NFAT-luc FcγRIIa-H effector cells. Antibodies were treated at 1 μg/mL and subsequently diluted at a 1:3 ratio until a final concentration of 0.152 ng/mL. Raji target cells were incubated with antibody and modified Jurkat NFAT-luc FcγRIIa-H effector cells for 6 h. Bio-Glo luciferase assay reagent was added to each well and luminescence was measured.

## Results

### Identification of candidate therapeutic targets on CD20 positive and negative B cells

**FIGURE 1:**
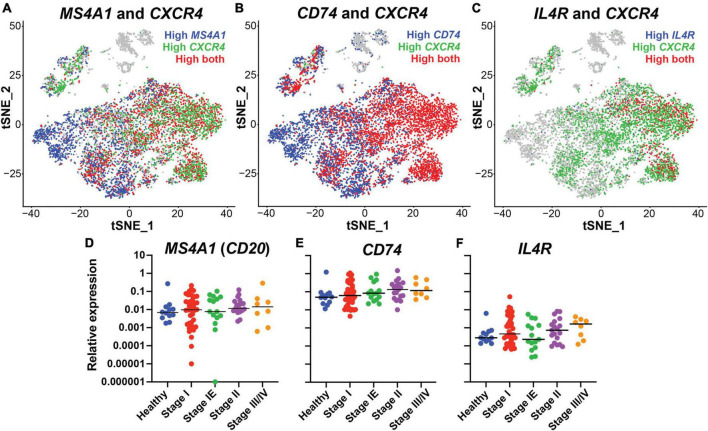
CD74 and IL4R identified as good candidates for bispecific antibody development, in conjunction with CD20, by single cell RNA sequencing. **(A)** tSNE plot of *MS4A1* (Blue) and *CXCR4* (Green). **(B)** tSNE plot of *CD74* (Blue) and *CXCR4* (Green). **(C)** tSNE plot of *IL4R* (Blue) and *CXCR4* (Green). **(D)** Relative expression of *MS4A1* in samples collected from healthy donors and donors with lymphoma at different stages of disease. Each dot represents one individual. **(E)** Relative expression of *CD74* in samples collected from healthy donors and donors with lymphoma at different stages of disease. **(F)** Relative expression of *IL4R* in samples collected from healthy donors and donors with lymphoma at different stages of disease. *n* = 12 healthy, 41 stage I, 15 stage IE, 19 stage II, 8 stage III/IV.

To identify putative B cell specific targets for the engineering of bispecific antibodies against DLBCL, we performed expression analysis of B cells isolated from healthy individuals using single cell RNA sequencing from a prior published study (SRA BioProject ID: PRJNA681021). CD20, the target of rituximab, is encoded by the *MS4A1* gene. Initial evaluation of the *MS4A1* levels showed heterogeneous expression within the B cell subpopulations (Figure 1A). CD20 and the chemokine receptor CXCR4 are critical for B cell trafficking and are molecular targets for cancer immunotherapies (34–37). Moreover, a prior study demonstrated that cells with high *CXCR4* expression were less responsive to rituximab treatment suggesting an inverse expression pattern (38, 39). Therefore, we examined the expression *CXCR4* and confirmed an inverse relationship between *CXCR4* and *CD20*, with a low Pearson correlation score of 0.008 (Figure 1A and Supplementary Table 1). CXCR4 is known to be expressed by many other immune cell types such as neutrophils and T cells, making it less specific therapeutic targeting of B cells (40–44). Thus, we focused on putative targets that correlated highly with *CXCR4* expression, were expressed on the B cell surface, and had FDA-approved monoclonal antibodies available. *CD74* and *IL4R* were identified as candidates for further investigation, with expression that overlapped with *CXCR4* and Pearson correlation scores of 0.325 and 0.307, respectively (Figures 1B,C and Supplementary Table 1). Additionally, *CD74* was broadly expressed and correlated highly with *MS4A1* expression while *IL4R* did not, with correlation scores of 0.523 and −0.064, respectively (Supplementary Table 1). Thus, both CD74 and IL4R could target B cells, even when CD20 is low or not present.

Next, we determined *MS4A1* (CD20), *CD74* and *IL4R* gene expression levels in 84 lymphoma samples across different tumor stages (stage I-III/IV) and compared to expression in 12 healthy tissue controls using quantitative PCR of tissue biopsies. We found that all three genes were expressed in lymphoma samples of all stages, with no significant difference compared to healthy control samples. *CD74* had the highest relative expression level followed by *MS4A1* and *IL4R*. There were no significant correlations with gene expression and tumor stage, although *CD74* expression did trend higher in later tumor stages (Figures 1D–F). These results demonstrated that with CD20, genes encoding CD74 and IL4R are highly expressed in lymphoma tissues, in addition to being broad B cell markers, and could represent candidate therapeutic targets for lymphoma.

### Engineering bispecific antibodies targeting CD74 or IL4R with anti-CD20

**FIGURE 2:**
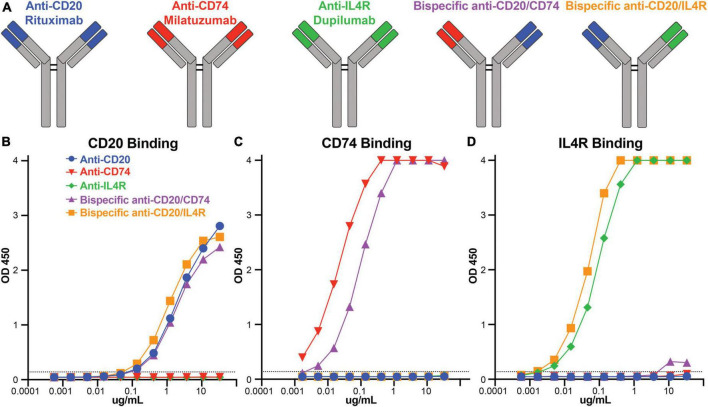
Bispecific antibody construction and antibody binding responses. **(A)** Schematic representation of rituximab, milatuzumab, dupilumab, and the bispecific antibodies constructed with anti-CD20/anti-CD74 heavy chains and anti-CD20/anti-IL4R heavy chains. **(B)** Line graph of OD450 absorbance obtained by ELISA measuring antibody binding to CD20 at serial dilutions. **(C)** Line graph of OD450 absorbance obtained by ELISA measuring antibody binding to CD74 at serial dilutions. **(D)** Line graph of OD450 absorbance obtained by ELISA measuring antibody binding to IL4R at serial dilutions. Background absorbance, represented by the dashed line, was considered 3X blank well OD450 measurement.

There are therapeutic antibodies that target CD74 (milatuzumab), IL4R (dupilumab), and CD20 (rituximab) that are approved for clinical use. We utilized these antibody combinations to engineer bispecific antibodies to combine anti-CD74 with anti-CD20 and anti-IL4R with anti-CD20 (Figure 2A). After bispecific antibody expression and purification, we determined antibody binding specificity against CD20, CD74, and IL4R recombinant protein antigens using ELISA. Using area under the curve (AUC) to compare binding response, anti-CD20 (rituximab) (AUC 78.1), bispecific anti-CD20/anti-CD74 (AUC 70.1) and bispecific anti-CD20/anti-IL4R (AUC 79.8) all bound to the recombinant CD20 with no appreciable non-specific binding from either anti-CD74 (milatuzumab) (AUC 1.6) or anti-IL4R (dupilumab) (AUC 1.5) antibodies (Figure 2B). Anti-CD74 (AUC 131.9) and bispecific anti-CD20/anti-CD74 (AUC 132.5) bound exclusively to CD74 with comparable levels of binding (Figure 2C). Likewise, anti-IL4R (AUC 132.6) and bispecific anti-CD20/anti-IL4R (AUC 133) bound exclusively and comparably to IL4R (Figure 2D). These data confirmed antibody specificity and similar binding levels between the monoclonal, single target antibodies and the bispecific antibodies.

### Anti-CD20 and anti-CD74-targeting antibodies mediate direct antibody-mediated cytotoxicity

**FIGURE 3:**
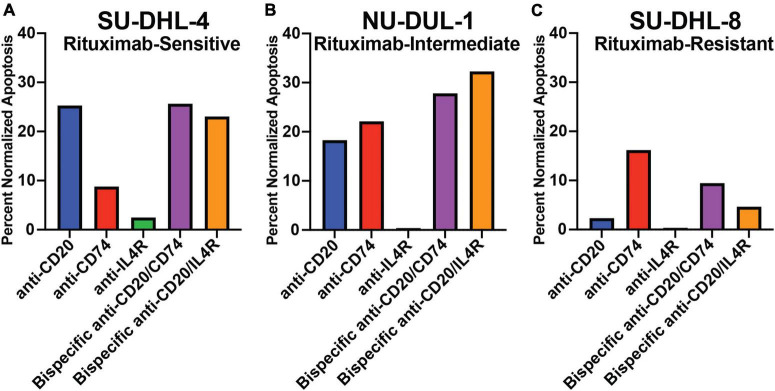
Apoptotic response to crosslinking antibodies within varying rituximab-resistant lymphoma cell lines, measured by annexin V staining. **(A)** Bar graph of the percent of total apoptosis within rituximab-sensitive, high CD20 expressing, SU-DHL-4 cells after 8 h of exposure to antibodies and crosslinking antibodies. **(B)** Bar graph of the percent of total apoptosis within rituximab-intermediate, intermediate CD20 expressing, NU-DUL-1 cells after 8 h of exposure to antibodies and crosslinking antibodies. **(C)** Bar graph of the percent of total apoptosis within rituximab-resistant, low CD20 expressing, SU-DHL-8 cells after 8 h of exposure to antibodies and crosslinking antibodies. All bar graphs normalized to respective cell line crosslinking antibody control.

We determined the ability of the antibodies to mediate direct cytotoxicity of three lymphoma cell lines by measuring apoptosis using annexin V in the presence of a cross-linking antibody. We utilized a rituximab-sensitive (SU-DHL-4), rituximab-intermediate (NH-DUL-1) and rituximab-resistant (SU-DHL-8) cell lines (38). First, we determined the expression of *MS4A*1, *CD74*, and *IL4R* in each cell line using qPCR (Supplementary Figure 2). *MS4A1* (CD20) gene expression was detected in all three cell lines but was reduced in the rituximab-intermediate and resistant cell lines (Supplementary Figure 2). *CD74* expression was detectable in all three cell lines, with no significant difference in expression level between the different cell lines. Similarly, *IL4R* was detectable in all three cell lines, and like *MS4A1*, was reduced in the rituximab-intermediate and resistant cell lines (Supplementary Figure 2). This data suggested that reduced gene expression of CD20 and IL4R in the NH-DUL-1 and SU-DHL-8 cell lines could contribute to the observed resistance to antibody therapy. Next, we measured antibody-mediated direct cytotoxicity of the cell lines. We found that the anti-CD20 antibody induced 25.5% apoptosis of the rituximab-sensitive SU-DHL-4 cell line (Figure 3A), 18.3 and 2.3% apoptosis of the intermediate (NH-DUL-1) and resistant (SU-DHL-8) cell lines, respectively (Figures 3B,C). Anti-CD74 antibody also induced direct cytotoxicity of SU-DHL-4 cell line at 8.6% and had higher percent cytotoxicity compared to rituximab (Figure 3A), of the NU-DUL-1 and SU-DHL-8 cell lines with 22.1 and 16.2% induced apoptosis, respectively (Figures 3B,C). Anti-IL4R antibody did not directly induce apoptosis in any of the tested cell lines with 2.7, 0.4, and 0.4% for the SU-DHL-4, NU-DUL-1 and SU-DHL-8 cell lines, respectively (Figures 3A–C). This data indicated that antibodies that are cross-linked to CD20 or CD74 could induce direct cellular cytotoxicity of lymphoma cells, whereas antibodies targeting IL4R could not. Moreover, reduced expression of CD20 on lymphoma cells reduced this cytotoxicity by anti-CD20 antibody.

The bispecific antibodies anti-CD20/anti-CD74 or anti-CD20/anti-IL4R could also mediate direct toxicity of the SU-DHL-4 cell line with 23.6 and 21.9% apoptosis, respectively, albeit not higher than anti-CD20 alone (25.5% apoptosis) (Figure 3A). However, the two bispecific antibodies had higher percent apoptosis of the rituximab-resistant cell lines NU-DUL-1 and SU-DHL-8 compared to anti-CD20 alone (Figures 3B,C). These results demonstrated that bispecific antibodies targeting CD20 and either CD74 or IL4R could mediate direct cytotoxicity of lymphoma cells, but when using a rituximab resistant lymphoma cell line (SU-DHL-8), bispecific anti-CD20/anti-CD74 or anti-CD74 alone induced the highest levels of apoptosis.

### Bispecific antibodies could mediate antibody-mediated cytotoxicity and phagocytosis of lymphoma antibody-dependent cellular cytotoxicity and antibody-dependent cellular phagocytosis

**FIGURE 4:**
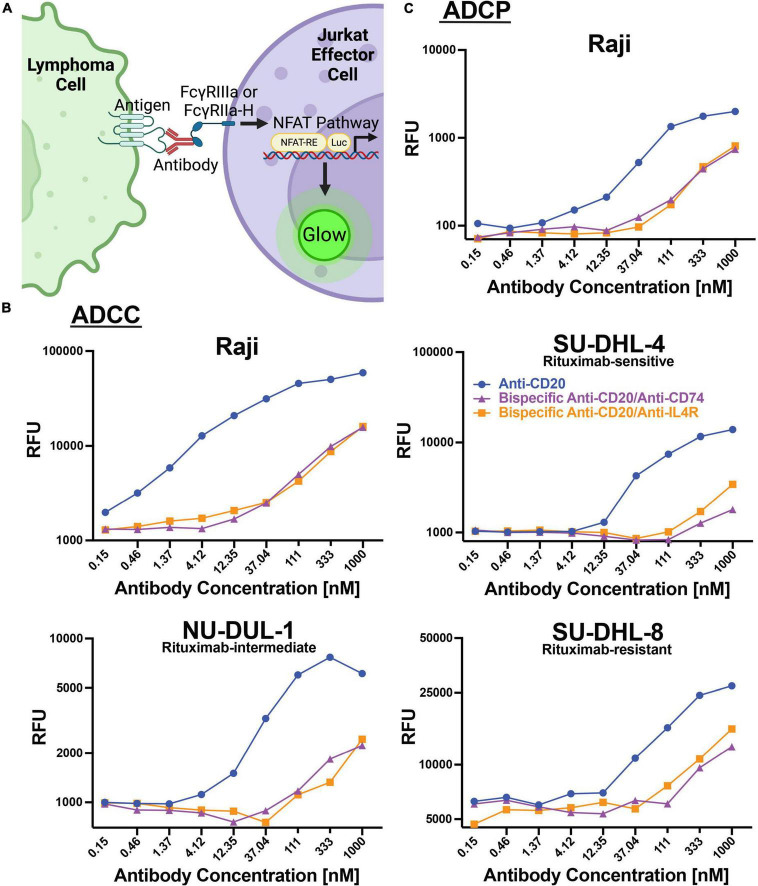
Antibodies induce ADCC and ADCP responses within lymphoma cell lines. **(A)** Schematic representation of the assays used to measure ADCC and ADCP response within modified Jurkat effector cells. Created with BioRender.com. **(B)** Line graphs of the relative fluorescent units (RFU) of luciferase produced by ADCC activation of FcγRIIIa modified Jurkat cells when incubated with Raji, SU-DHL-4, NU-DUL-1, or SU-DHL-8 DLBCL cell lines and serial diluted anti-CD20 (Blue), bispecific anti-CD20/anti-CD74 (Purple), or bispecific anti-CD20/anti-IL4R (Orange) antibodies. **(C)** Line graph of the relative fluorescent units (RFU) of luciferase produced by ADCC activation of FcγRIIa-H modified Jurkat cells when incubated with Burkitt’s lymphoma Raji cells and serial diluted anti-CD20 (Blue), bispecific anti-CD20/anti-CD74 (Purple), or bispecific anti-CD20/anti-IL4R (Orange) antibodies.

In addition to direct cytotoxicity of lymphoma cells by engaging cellular receptors, therapeutic antibodies can engage and recruit effector cells through Fc-Fc receptor interactions to mediate antibody-dependent cellular cytotoxicity (ADCC) or ADCP. We utilized a cell-based assay employing a reporter gene that generates luciferase downstream of the Fc receptor pathway (Promega). For ADCC, FcγRIIIa is engaged and for ADCP, FcγRIIa-H is primarily engaged (Figure 4A). We utilized these cell lines as proxies for ADCC and ADCP activities. We found that anti-CD20 alone could mediate ADCC with increasing concentrations of antibody using Raji (AUC 50,712), SU-DHL-4 (11,125), NU-DUL-1 (AUC 6,535), and SU-DHL-8 (AUC 22,929) lymphoma targets (Figure 4B). The two bispecific antibodies (anti-CD20/anti-CD74 and anti-CD20/anti-IL4R) could also induce ADCC, but at lower levels than anti-CD20 alone. Anti-CD20/anti-CD74 bispecific ADCC AUC for Raji, SU-DHL-4, NU-DUL-1 and SU-DHL-8 cell targets were: 10,512, 1,351, 1,798, and 9,815, respectively (Figure 4B). Anti-CD20/anti-IL4R bispecific ADCC AUC for Raji, SU-DHL-4, NU-DUL-1 and SU-DHL-8 cell targets were: 10,001, 2,123, 1,623, and 11,610, respectively (Figure 4B). Similarly, anti-CD20 and the bispecific antibodies could induce ADCP of Raji cell targets using our cell reporter system, with anti-CD20 having a higher magnitude of response (Figure 4C). The AUC for anti-CD20, bispecific anti-CD20/anti-CD74, and bispecific anti-CD20/anti-IL4R being 1,679, 482, and 510, respectively. These data showed that the anti-CD20 monoclonal antibody and both bispecific antibodies containing anti-CD20 could induce ADCC and ADCP immune responses, although the bispecific antibodies had lower magnitude of ADCC and ADCP at the same antibody concentrations.

## Discussion

Although therapeutic applications of bispecific antibodies are relatively new, the dual nature of the antibodies could be predicted to decrease the ability of lymphomas to escape treatment or develop resistance as two cellular targets would need to be escaped. Besides tagging cells for destruction, bispecific antibodies may also be designed to deliver nanoparticles or drugs to target cells (45). Additionally, some treatments such as CAR-T cells require significant modifications specific to each patient, a costly and time-consuming series of steps that may be avoided with the use of bispecific antibodies (46, 47). Here, we investigated the ability of anti-CD20/anti-CD74 and anti-CD20/anti-IL4R bispecific antibodies to bind their designated targets on lymphoma cells and to kill their targeted cells *via* different mechanisms.

Using B cells isolated from healthy patients, we explored other potential B cell markers that previously have FDA-approved antibody therapies available and focused on CD74 and IL4R. CD74, a transmembrane glycoprotein that functions as a survival receptor, is highly expressed in malignant B cells (48, 49). Stein et al. demonstrated survival of immune-deficient mice with lymphoma was significantly improved when treated with anti-CD74 antibody, especially when administered in conjunction with rituximab (50). Milatuzumab, the first anti-CD74 monoclonal antibody approved by the FDA for clinical practice, is effective at treating aggressive B cell malignancies such as multiple myeloma, especially in combination with rituximab (49–51). When PBMC samples from patients were treated with milatuzumab, naïve and memory B cells were bound by milatuzumab 98.3 and 97% of the time, respectively (52). Although IL4R expression was limited to a smaller population of total B cells, it provided an opportunity to target cells otherwise capable of escaping rituximab treatment due to their low CD20 expression. Cells treated with the IL4 antagonist, APG201, were more susceptible to chemotherapeutics, suggesting a role for IL4 pathway signaling in chemotherapy resistance (53). Furthermore, anti-IL4R treatments reduced inflammatory cell recruitment, improved measurable lung function and decreased overall asthma symptoms in both humans and monkeys (54, 55). Consequently, both targets would help expand the range of targeted cells compared to current rituximab treatment.

The bispecific antibodies, anti-CD74/anti-CD20 and anti-IL4R/anti-CD20, caused significantly more apoptosis than anti-CD20 alone in both the rituximab intermediate (NU-DUL-1) and rituximab resistant (SU-DHL-8) cell, while the rituximab-sensitive (SU-DHL-4) cell line showed comparable levels of apoptosis between the bispecific antibodies and anti-CD20. These data demonstrate the potential benefit of using bispecific antibodies in conditions of rituximab resistance without losing significant apoptosis induction in conditions of continued rituximab sensitivity. It is interesting to note that the relative patterns of apoptosis induction by anti-CD74 across the cell lines appear to be negatively correlated with CD20 expression: the rituximab-resistant cell line exhibited higher killing by anti-CD74 compared to anti-CD20, while the rituximab-sensitive cell line exhibited lower apoptosis by anti-CD74. Milatuzumab, which has previously been shown to reduce cell growth and proliferation of B cells (52), mediated direct apoptosis of the lymphoma cells in this study. The anti-IL4R drug, dupilumab, an antagonist for the IL4 signaling pathway (56, 57), did not robustly elicit cytotoxicity here. This highlights both CD20 and CD74 as targets to facilitate direct killing of lymphoma cells without the need for effector cells. The significant utility of bispecific antibodies is demonstrated here as anti-IL4R treatment only was quite ineffective, while using an anti-CD20/anti-IL4R bispecific antibody caused apoptosis significantly higher than—or at least comparable to—the anti-CD20 treatment in all cell lines. Gupta et al. presented similar results in other bispecific antibodies, with very little apoptosis after treatment with monoclonal antibodies for CD20 or CD74, but anti-CD20/anti-CD74 bispecific antibodies displayed 3–4 times higher apoptosis than either monoclonal antibody alone (58).

ADCC experiments revealed high activity with anti-CD20 treatment, though the bispecific antibodies showed decreased activity in all cell lines. The decrease in ADCC of the bispecific antibodies could be due to the having only a single Fab arm of the antibody for each target resulting in reduced antibody affinity or other binding kinetic attributes. The decreased effectiveness of the bispecific antibody containing anti-CD74 may also be due to the rapid internalization causing difficulty for the effector cells to detect cell surface antibodies, therefore making cell recruitment unlikely (59–61). Stein et al. explicitly demonstrated that anti-CD74 antibody treatment did not produce significant ADCC in Raji cells when cocultured with purified human leukocyte populations from peripheral blood samples (50). Here, ADCC experiments utilized only T-lymphocytes, however, it is possible that ADCC *via* NK cells would occur with anti-CD74 treatment (62, 63). ADCP activity was high in Raji cells when treated with anti-CD20, with both anti-CD20/anti-CD74 and anti-CD20/anti-IL4R bispecific antibodies having lower than anti-CD20 but still elevated ADCP response. These bispecific antibodies have shown to be effective in all methods of cell killing evaluated, similar to those results of anti-CD20 alone.

Bispecific antibodies are already being tested in clinical trials and are demonstrating their utility in a broad range of diseases (64–68). The bispecific anti-CD20/anti-CD74 antibody has been tested in mantle cell lymphoma, an NHL sub-type and has significantly improved survival of mice with lymphoma (58). Furthermore, an anti-IL4Rα/anti-IL5 bispecific antibody decreased the number of recruited lymphocytes and eosinophils during asthmatic reactions in mice more effectively than when either antibody was administered alone or concurrently, demonstrating the ability of IL4R bispecific antibodies to be effective in alleviating symptoms of asthma (54). Finally, an anti-CD20/anti-CD3 bispecific antibody targeted lymphoma and cytotoxic T-cells, showed high killing capacity both *in vivo* and *in vitro*, even with very low cell surface expression of CD20; anti-CD20 alone was unable to cause significant cell death (46). Phase I clinical trials of this anti-CD20/anti-CD3 antibody, Glofitamab, showed that at high doses, nearly 50% of previously treated patients with aggressive NHL had complete recovery after treatment, and 81% of those remained disease free past 2 years following treatment (47). Previous reports in mantle cell lymphoma cell lines show distinct cytoskeletal dynamics, ROS generation, and disruptions of NF-κB pathways after treatment with rituximab and milatuzumab (51). Future studies would benefit from the use of single-cell RNA sequencing to identify unique downstream pathways of cell death induced by these bispecific anti-CD20/anti-CD74 and anti-CD20/anti-IL4R antibodies.

There were several key limitations of our study that should be addressed in future work. One major limitation is that we evaluated the function of the antibodies in *in vitro* model systems. While these models could identify promising therapeutic targets and antibodies, *in vivo* evaluation of the antibodies in animal models will confirm the therapeutic efficacy of these bispecific antibodies. Moreover, *in vivo* studies will be needed to determine any off-target, and other toxicities, that could be caused by these antibodies. Studies *in vivo* will also be required to determine the pharmacokinetics of these antibodies in order to determine the clinical feasibility of using them for treatment. Lastly, we evaluated cytotoxicity of several lymphoma cell lines that may not represent primary tumors of broad types. Further study with primary tumors or other types of B-cell derived tumors will be required.

In summary, bispecific antibodies targeting CD74 or IL4R could mediate ADCC and ADCP, while anti-CD74 targeting antibodies could mediate direct cellular cytotoxicity similar to anti-CD20. This observation, coupled with higher expression of CD74 on lymphoma cells, leads to anti-CD74 and anti-CD20 immunotherapies as better therapeutic targets. To further develop these bispecific antibodies as a potential future therapeutic, it would be beneficial to evaluate the level of acquired resistance that develops with the use of prolonged bispecific antibodies both *in vitro* and *in vivo*. These data demonstrate that the dual specificity of engineered bispecific antibodies is an effective future prospect of cancer immunotherapy.

## Data availability statement

The original contributions presented in this study are included in the article/Supplementary material, further inquiries can be directed to the corresponding author. The single-cell RNA Sequencing Data SRA BioProject ID: PRJNA681021.

## Author contributions

TB and EG: conceptualization. EG, SAK, and SP: laboratory experimentation. EG and SK: data analysis. TB, RM, SAK, and EG: writing. All authors contributed to the article and approved the submitted version.
