# Supplementary material for: Development of combinatorial antibody therapies for diffuse large B cell lymphoma
Source: Front Med (Lausanne). 2022 Oct 24;9:1034594. doi: 10.3389/fmed.2022.1034594 (PMC9637670; doi:10.3389/fmed.2022.1034594)
Supplement: Supplementary file 2 [file Data_Sheet_1.pdf]

SUPPLEMENTAL INFORMATION

Development of combinatorial antibody therapies for diffuse large B cell lymphoma

Eric S. Geanes, Stacey A. Krepel, Rebecca McLennan, Stephen Pierce, Santosh Khanal, and Todd Bradley.

Table of contents

Supplemental Data 1- Sequences used for heavy and light chains in each of the bispecific antibodies.	Page 2
Supplementary Figure 1- Quality control for custom bispecific antibodies	Page 3
Supplementary Figure 2- Gene expression determined by qPCR for each cell line.	Page 4
Supplementary Table 1- Pearson correlations of gene expression in B cells	xlsx file

Supplemental Data 1- Sequences used for heavy and light chains in each of the bispecific antibodies.

Antibody Sequences

Rituximab

Variable Heavy Chain

QVQLQQPGAELVKPGASVKMSCKASGYTFTSYNMHWVKQTPGRGLE
WIGAIYPGNGDTSYNQKFKGKATLTADKSSSTAYMQLSSLTSEDSAVY
YCARSTYYGGDWYFNVWGAGTTVTVSA

Variable Light Chain

QIVLSQSPAILSASPGEKVTMTCRASSSVSYIHWFQQKPGSSPKPWIYA
TSNLASGVPVRFSGSGSGTSYSLTISRVEAEDAATYYCQQWTSNPPTF
GGGTKLEIK

Milatuzumab

Variable Heavy Chain

QVQLQQSGSELKKPGASVKVSCKASGYTFTNYGVNWIKQAPGQGLQ
WMGWINPNTGEPTFDDDFKGRFAFSLDTSVSTAYLQISSLKADDTAVY
FCSRSRGKNEAWFAYWGQGTLVTVSS

Variable Light Chain

DIQLTQSPLSLPVTLGQPASISCRSSQSLVHRNGNTYLHWFQQRPGQS
PRLLIYTVSNRFSGVPDRFSGSGSGTDFTLKISRVEAEDVGVYFCSQSS
HVPPTFGAGTRLEIK

Dupilumab

Variable Heavy Chain

EVQLVESGGGLEQPGGSLRLSCAGSGFTFRDYAMTWVRQAPGKGLE
WVSSISGSGGNTYYADSVKGRFTISRDNSKNTLYLQMNSLRAEDTAVY
YCAKDRLSITIRPRYYGLDVWGQGTTVTVSS

Variable Light Chain

DIVMTQSPLSLPVTPGEPASISCRSSQSLLYSIGYNYLDWYLQKSGQSP
QLLIYLGSNRASGVPDRFSGSGSGTDFTLKISRVEAEDVGFYYCMQAL
QTPYTFGQGTKLEIK

A

Anti-CD20 (Rituximab)/anti-CD74 (Milatuzumab) Bispecific Antibody

Reducing SDS-PAGE

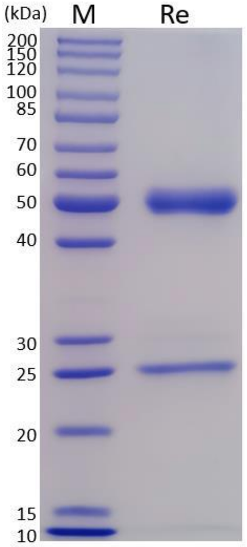

Size exclusion chromography-HPLC

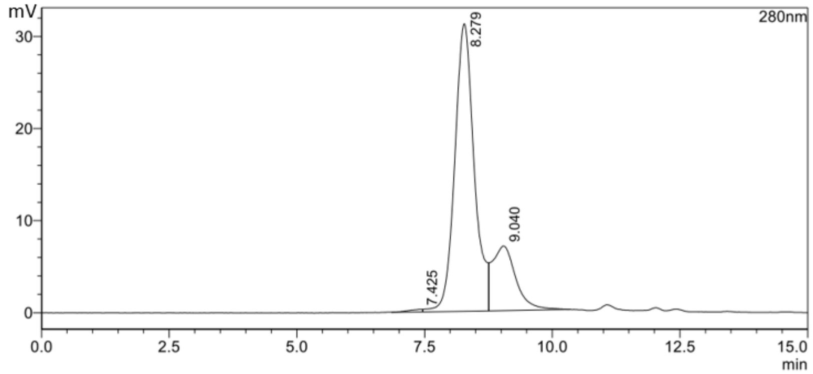

	Time (min)	Area	% Area
1	7.425	5707	0.541
2	8.279	825066	78.200
3	9.040	224303	21.259

B

Anti-CD20 (Rituximab)/anti-IL4R (Dupilumab) Bispecific Antibody

Reducing SDS-PAGE

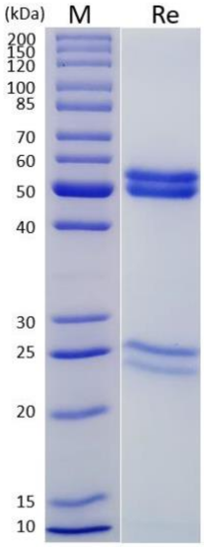

Size exclusion chromography-HPLC

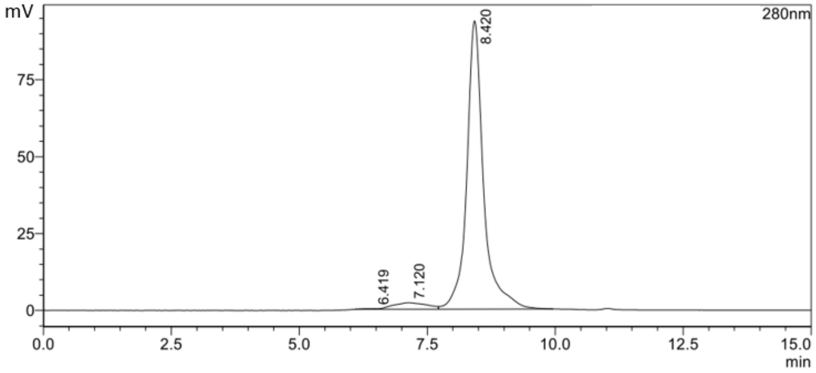

	Time (min)	Area	% Area
1	6.419	2799	0.125
2	7.120	95631	4.275
3	8.420	2138697	95.600

Supplementary Figure 1- Quality control for custom bispecific antibodies. (**A**) Reducing SDS-PAGE gel and size exclusion chromatography for anti-CD20/anti-CD74 bispecific antibody. (**B**) Reducing SDS-PAGE gel and size exclusion chromatography for anti-CD20/anti-IL4R bispecific antibody.

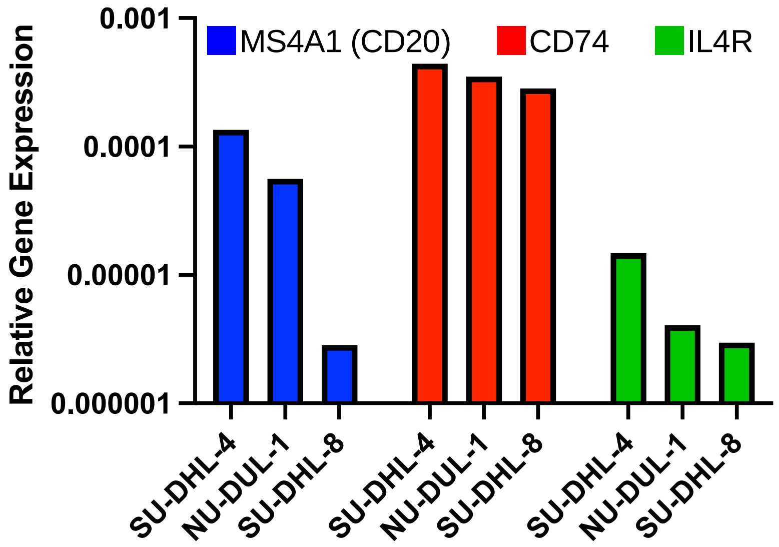

Supplementary Figure 2- Expression of *MS4A1*, *CD74*, and *IL4R* in the three lymphoma cell lines. Expression of each gene determined by quantitative PCR using probes that target *MS4A1*, *CD74* and *IL4R* using RNA extracted from the three lymphoma cell lines. Expression of each gene is relative to the control gene expression of 18S.
